# Decision support tools in the absence of equipoise: the case for vaccination

**DOI:** 10.1136/bmjph-2025-003789

**Published:** 2025-11-04

**Authors:** Daphne Bussink-Voorend, Marlies EJL Hulscher, Glyn Elwyn

**Affiliations:** 1 Radboud Institute for Medical Innovation, Primary and Community Care, Radboud University Medical Center, Nijmegen, Netherlands; 2 Radboud Institute for Medical Innovation, IQ Health Science Department, Radboud University Medical Center, Nijmegen, Netherlands; 3 The Dartmouth Institute for Health Policy and Clinical Practice, Geisel School of Medicine at Dartmouth College, Lebanon, New Hampshire, USA

**Keywords:** Public Health, Vaccination, Public Health Practice

 Public health is in a bind. The goal of the discipline is to protect and improve the public’s overall health so that populations remain as healthy as possible. Public health considers decisions from a Malthusian perspective: what is good for the overall population and often sidesteps the issue of personal choice, and the extent to which that choice is informed or not. What should public health do about individuals who, informed or otherwise, act in ways that threaten their health or that of others, for instance, by smoking, declining screening and refusing vaccinations?

Individual behaviour should be understood within a broader context, where many factors and societal developments drive behaviours such as smoking and vaccine refusal. For example, decision-making about vaccination is shaped by a perceived need to vaccinate, concerns about safety, social networks and norms, past experiences with healthcare services and institutional trust. Vaccine refusal is highly context dependent.^1^ Therefore, vaccination cannot be viewed solely as an individual health choice. At the same time, this should not stop us proposing interventions targeted at individuals as part of broader public health strategies. Vaccination is ideally suited for this purpose, as choices made by individuals impact others through the protective effect of group immunity.

In healthcare, tools are being increasingly developed to support individual decision-making, particularly when comparing reasonable treatment options to assess benefit-harm trade-offs. These tools come in various formats, ranging from brief comparison tables to more comprehensive web applications. They are designed to support shared decision-making between healthcare professionals and their patients, which leads to better patient knowledge, better risk perceptions, and greater clarity about personal preferences.^2^ Moreover, decision support tools are typically developed in co-creation with end-users to increase acceptability, usability and relevance.

Public health information efforts have largely avoided this comparative approach, instead providing recommendations rather than comparing whether to accept or reject an intervention and involving community voices.^3^ Is it possible to advocate for the use of client-facing decision support tools in public health?

Shared decision-making is ideal when multiple options are reasonable, a situation termed equipoise. The guidelines established by the International Patient Decision Aid Standard collaboration advocate a balanced representation of options. Yet, it is also acceptable to use a shared decision-making approach in situations where options are not entirely balanced.^4^ For example, in the case of vaccination, broader societal interests are relevant alongside individual preferences.^5^ Evidence supports uptake, because the risk of harm is very low compared with the benefits of avoiding illness. Therefore, when there is clear evidence of population-level benefits, developers of decision support tools should strive for a different kind of balance that reflects the evidence. They should be clear about the overwhelming benefits to both individuals and populations, while also being equally clear about the very low risk of harm. Alternative criteria are needed to guide the design of decision support for situations where broader interests need to be considered, as well as the preponderance of evidence.

Emphasising personal choice can be at odds with public health’s emphasis on the overall population. Yet, vaccination coverage in a given population is the result of multiple individual choices. Because the consequences of these individual vaccination decisions also impact a wider society, it is in the public interest to support these choices with trustworthy information. This is especially true when people make choices in the face of pervasive misinformation and disinformation and the politicisation of public health topics.^6 7^ The decline in vaccine uptake rates and the resurgence of vaccine-preventable diseases highlight the high relevance of this topic.^8^

In times of fear and confusion, it is not only important to clearly communicate the benefits and harms of vaccination, but also to listen and respect other people’s views. Approaching vaccination as a strict medical consideration does not do justice to the relevance of social processes, institutional trust and political trends.^1^ Considering how these factors shape individual concerns about vaccination improves our understanding of how to relate to vaccine-hesitant individuals and which factors drive acceptance. An open dialogue about concerns, emotions and values, which is central to shared decision-making, is a good starting point. Logically, we need to design tools that support this process. Decision support tools about vaccination should also reflect deep community engagement, political awareness and cultural sensitivity to build trust and ensure their relevance. This is best accomplished through collaboration in multidisciplinary teams of community members, clinicians, biomedical and social scientists and public health professionals.

Our understanding of how to design decision support tools has evolved, resulting in a body of knowledge about their practical use. We should also harness this potential to ethically promote public health interventions where it is essential to balance societal interests alongside respect for individual choice.

## Data Availability

Data sharing not applicable as no datasets generated and/or analysed for this study.
